# Perforation of the Antrum Maxillare at the Base of the Malar Process

**Published:** 1848-10

**Authors:** W. H. Dwinelle


### ARTICLE V.

### *Perforation of the Antrum Maxillare at the Base of the Malar Process.* By W. H. DWINELLE, D. D. S.

The operation of puncturing the antrum maxillare, in treatment of abscesses located there, has become so common, and has been so repeatedly detailed in the Journal, that I should feel unjustified in referring to the case now under consideration, were it not for a series of novelties that seem somewhat singularly associated with it.

About two years age, Mrs. A——, of our place, aged about fifty years, called at my office, and had all of her superior teeth removed, some eight or ten in number, they all being more or less diseased. Nothing unusual attended the operation, except on the extraction of the first molar on the left side; this was accompanied with considerable pain; its roots were unusually long, their periosteum exceedingly inflamed, and to the extremity of each was attached an abscess. The teeth had heretofore caused her such constant and extensive pain, that her health had become so impaired as to render her a confirmed invalid; pains of the most excruciating character were constantly darting through the left side of the face, the eye, through the temporal region, and around to the upper part of the occipital bone, rendering all of these parts, for the most of the time, exceedingly sensitive even to the touch. The operation afforded her immediate relief.

The gums healed up readily; and about eight months afterwards, she applied to me for a full upper set of artificial teeth. I found the alveolar ridge apparently permanently absorbed; but when I touched the parts corresponding to the locality of the diseased molar, I found it quite tender, although it appeared, externally, as healthy as the adjacent parts. I deemed it prudent to defer taking an impression, for a few weeks, the other symptoms in the meantime having fully subsided.

About three months afterwards I completed her a full upper set of artificial teeth, to be sustained by atmospheric pressure. These she wore with ease for nearly a year, during which time her health continued greatly to improve. Not seeing her for several months, I had concluded she had become perfectly restored; when she called one day, and informed me that all of her old symptoms had returned, together with the additional ones, of a great sense of internal pressure from "under the cheek bone," a sense of heaviness and fullness there, and a feeling as though the left side of the nose was closed; the eye seemed pressed and protruded from its socket, while a continual caustic excoriating secretion was constantly dropping down the nasal glands into the throat. The palatine arch immediately under the antrum, had become so exceedingly sensitive, although its appearance was perfectly healthy, that it could not endure the pressure of the atmosphere necessary to sustain the plate. These symptoms had been gradually coming on for about two months.

I at once concluded that an abscess within the antrum was the cause of her troubles, and proposed operating, after explaining to her the nature and probable results of such a course, she finally consented to an operation.

The superior teeth being all gone, and the parts fully consolidated, of course I could not adopt the usual mode of operating. Having completed my arrangements, with a strong, sharp scalpel, I made two incisions, one across the other, at the base of the *malar process*, about six lines above the alveolar ridge, at a point immediately above the position of the diseased molar tooth. I then dissected up the four angles from the bone, so as to leave a free space about four lines in diameter; after which I placed a small acute-pointed drill upon the bone I had uncovered, directing it upward and inward, I drilled into the cavity of the antrum. On withdrawing the instrument, I found it covered with a thick, dark yellow and fetid pus. Then, with a cone-shaped reamer, I enlarged the orifice to the diameter of about three lines; a small quantity of matter followed this as it was removed. I then passed a probe into the orifice, and carried it up so far as to reach the orbital surface. After taking the usual precautions to keep the orifice open, I dismissed the patient until the following day, when I syringed out the cavity with a weak solution of nitrate of silver; this I continued to do daily, until the cure was completed.

The operation immediately dispelled all of the unpleasant symptons enumerated above; it was also succeeded by a feeling of relief she had not experienced for years. The pressure was immediately taken off the eye; the singular pain in the back part of the head ceased; and the discharge through the nasal glands was no longer a source of annoyance.

It is now about four weeks since I suffered the orifice leading to the cavity to close; since then there has not been the least indication of a return of any of the old symptoms.

It is somewhat remarkable that the palatine bone immediately under the affected part, has materially changed its shape, occasioning a depression in the direction of the antrum; so that the set of teeth already made, will have to be entirely remodeled, before they can be adapted again to the mouth. I shall be in no haste to do this, until I am satisfied that the parts have assumed a permanent position.

There can be no doubt but that the disease of the antrum in this instance, had existed for a long time; and that it was originally communicated to it by the diseased molar, whose extremities, in all probability, extended entirely into it.

I trust the relation of the case may prove as profitable to the readers of the Journal, as its experience has been to me. I think I can discover a gentle admonition in its teachings, not to be too hasty under like circumstances again; and in every instance to fully acquaint myself with all of the previous symptoms and peculiarities of cases of disease or deformity of the mouth, before I attempt their correction.

